# DNA methylation integratedly modulates the expression of Pit-Oct-Unt transcription factors in esophageal squamous cell carcinoma

**DOI:** 10.7150/jca.49231

**Published:** 2021-01-15

**Authors:** Wei He, Shuai Gong, Xin Wang, Xinhua Dong, Hua Cheng

**Affiliations:** 1 Department of Oncology, the First Affiliated Hospital of Zhengzhou University, Zhengzhou 450052, Henan province, China.; 2 Department of Radiotherapy, the First Affiliated Hospital of Zhengzhou University, Zhengzhou 450052, Henan province, China.; 3 Department of Gastrointestinal Surgery, the First Affiliated Hospital of Zhengzhou University, Zhengzhou 450052, Henan province, China.; 4 Department of Oncology, Xiayi Hospital of Traditional Chinese Medicine. Shangqiu 476400, Henan province, China.

**Keywords:** OCT transcription factor, DNA methylation, esophageal squamous cell carcinoma, clinical characteristics

## Abstract

**Background:** Dysregulation of Pit-Oct-Unc family transcription factors has been implicated in esophageal squamous cell carcinoma (ESCC). In this study, we evaluated the expression and promoter methylation status of Octamer (OCT) transcription factor genes in human ESCC clinical specimens to investigate the mechanism underlying this observation along with the clinical significance.

**Methods:** Total DNA or RNA was extracted from ESCC tissue specimens and the mRNA level of genes encoding the transcription factors OCT1, OCT2, OCT3/OCT4, OCT5, OCT7, OCT9, and OCT11 were evaluated by quantitative PCR. The DNA methylation status of gene promoters was assessed by bisulfite pyrosequencing and next-generation sequencing. The relationship between the expression of these transcription factors and ESCC proliferation was investigated *in vitro* and *in vivo* with the colony formation assay and a mouse xenograft tumor model, respectively. We also examined the correlation between *OCT* gene expression and promoter methylation and clinicopathologic characteristics of ESCC.

**Results:** *OCT1* was upregulated whereas *OCT4*, *OCT6*, and *OCT11* were downregulated in ESCC compared to non-tumor tissue. *OCT2*, *OCT7*, and *OCT9* were undetected in all samples. *OCT1*, *OCT6*, and *OCT11* levels were negatively correlated with the methylation of their respective promoters, but there was no relationship between *OCT4* expression and promoter methylation status.

**Conclusion:** Changes in promoter methylation rate underlie the observed alterations in *OCT1*, *OCT6*, and *OCT11* expression in ESCC, whereas another mechanism is likely responsible for the dysregulation of *OCT4.*

## Introduction

Pit-Oct-Unc (POU) family transcription factors mediate the transcription of downstream genes that are necessary for the maintenance of pluripotency in embryonic stem cells but also promote the proliferation or stemness of human cancers by binding to the octamer sequence motif (AGTCAAAT consensus sequence) of gene promoters. Octamer (OCT) transcription factors belonging to the POU family have been shown to play a regulatory role in cancer cells 1-3. However, their functions in esophageal squamous cell carcinoma (ESCC) are not known. ESCC is one of the most fatal malignancies of the digestive system; the incidence of ESCC is especially high in certain regions such as Linxian, Anyang City, Henan Province, China 4-6. Clarifying the expression patterns of OCT proteins in ESCC can provide insight into their functions, which can in turn serve as a basis for the development of more effective diagnostic and therapeutic strategies.

Methylation of gene promoters is an epigenetic modification that regulates gene transcription 7, 8. It was previously reported that the expression of OCT transcription factors is dysregulated in ESCC 9, 10. In order to elucidate the mechanism underlying this observation, in the present study we examined the expression levels of OCT family proteins in ESCC tissue samples and the methylation status of their gene promoters, as well as the relationships between these two variables and their association with clinicopathologic features of ESCC.

## Material and Methods

### Patients and Clinical samples

A series of 150 surgically resected fresh ESCC and their corresponding adjacent normal tissue samples were collected at the First Affiliated Hospital of Zhengzhou University (Zhengzhou, China) and snap-frozen in liquid nitrogen from 2012 to 2016 (Supplemental table 1). No patients had received any preoperative treatment. Among them, 98 are men and 52 are women, ranging from 37-76 years of age with a mean age of 58.2 years. According to the International Union against Cancer (UICC) 2017 standard, 5 cases were classified as stage I, 39 were stage II, 97 were stage III and 9 were stage IV. Histologically, 52 were well differentiated, 58 were moderate and 40 were poorly differentiated. The collection of clinical specimens and all experiments were performed according to the Helsinki Declaration and were reviewed and approved by the ethics committee of the First Affiliated Hospital of Zhengzhou University, Zhengzhou City, Henan Province, China.

### Cell lines and agents

We used three ESCC cell lines (KYSE70, KYSE140, and KYSE450) that have also been described in our previous work 10. Patient-derived cells were prepared from ESCC tissue specimens according to a published protocol 11. Het-1A human esophageal epithelial cells were preserved in our laboratory. Lentivirus particles harboring the full-length cDNA sequence of OCT or two small interfering RNA (siRNA) targeting OCT1 (siRNA-1 or siRNA-2 of OCT1) were prepared by Vigene Corporation (Jinan City, China). The anticancer agents cisplatin, 5-fluorouracil (5-FU), and paclitaxel were purchased from Selleck Chemicals (Houston, TX, USA) and dissolved in dimethylsulfoxide (DMSO) for *in vitro* experiments and in DMSO along with polyethylene glycol 400 or Tween 80 for use in mice. The agents were prepared according to a published protocol 12.

### Bisulfite sequencing (BSP) and next-generation sequencing (NGS) 13

Genomic DNA was extracted from ESCC or non-tumor tissue using the QIAamp DNA Mini Kit (Qiagen, Hilden, Germany) according to the manufacturer's protocol. DNA concentration and quality were determined using a spectrophotometer (Thermo Fisher Scientific, Waltham, MA, USA). The samples were subjected to bisulfite treatment with the ZYMO EZ DNA Methylation-Gold Kit (Zymo Research, Orange, CA, USA) according to the manufacturer's protocol. The promoter region of each target gene - i.e., the sequence 2000 bp upstream of the transcription start site—was obtained by searching the NCBI database. Methyl Primer Express v1.0 software (Thermo Fisher Scientific) was used to identify and predict CpG sites in the promoter sequence and primers were designed for PCR amplification of this region. The amplified product, which was about 218 bp in size, was purified using magnetic beads for sequencing library construction (Life Technologies, Carlsbad, CA, USA), and a barcode was added for second-generation sequencing on the Iontorrent PGM platform (Life Technologies). If the sequencing result of the OCTs' promoter region after BSP treatment is C, it indicates that the CpG site was methylated; if the sequence of the OCTs promoter region sequencing after BSP treatment was T, it indicated that the methylation of the CpG site was not occurred. The methylation rate of OCT gene promoter sequences was calculated as the number of methylated CpG sites divided by the total number of CpG sites. The forward and reverse primers used for BSP and NGS experiments were as follows: Oct1/POU2F1, 5'-ATTGAGGGYGTTGTTTTAGTT-3' and 5'-CCTCAAAAAAACTCCACC-3'; Oct4/POU5F1, 5'-GTGGTTAGGTATTTTGGGAGGT-3' and 5'-CAAACTAAACTCR AACTCCC-3'; Oct6/POU3F1, 5'-TYGAGATTTTTTTTTTTTGGAATT-3' and 5'-AACRA TTCTACAATCCTACRC-3'; and Oct11/POU2F3, 5'-TTGTAATTTTAGGGAAG TTTAATTGA-3' and 5'-CTCAAATTCTCTTATCCCTAATTAAA-3' (where R = A or G; Y = C or T).

### Quantitative polymerase chain reaction (qPCR)

RNA was extracted from paired ESCC and non-tumor clinical specimens using the PARIS Kit (Thermo Fisher Scientific) and reverse transcribed using Multiscribe reverse transcriptase (Thermo Fisher Scientific) according to the manufacturer's protocol. qPCR was performed as previously described 14, 15 to analyze the expression levels of *OCT1*, *OCT2*, *OCT3*/*OCT4*, *OCT5*, *OCT7*, *OCT9*, and *OCT11*. The forward and reverse primers used for qPCR were as follows: Oct1/POU2F1, 5'-GAAACGCACCAGCATAGAGACC-3' and 5'-GGCGGTTACAG AACCAAACACG-3'; Oct2/SLC22A2, 5'-GAGATAGTCTGCCTGGTCAATGC-3' and 5'-GTAGACCAGGAA TGGCGTGATG-3'; Oct4/POU5F1, 5'-CCTGAAGCAGAAGA GGATCACC-3' and 5'-AAAGCGGCAGATGGTCGTTTGG-3'; Oct6/POU3F1, 5'-GTGTTC TCGCAGA CCACCATCT-3' and 5'-CGCGA TCTTGTCCAGGTTGGTG-3'; Oct7/POU3F2, 5'-GTGTTCTCGCAGACCACCATCT-3' and 5'-GCTGCGATCTTGTCTATGCTCG-3'; Oct9/POU3F4, 5'-GTGTTCTCGCAGACCACCATCT-3' and 5'-GCGATCTTGTCAAT GCTGGTCG-3'; and Oct11/POU2F3, 5'-GCTGGAGAAGTTTGCCAAGACC-3' and 5'-GTGAGATGGTGGTCTGGCTGAA-3'. The *β-Actin* gene (5' CACCATTGG CAATGAGCGGTTC-3' and 5'- AGGTCTTTGCGGATGTCCACGT-3') was used as an internal control for calculating the relative expression level of target genes.

### Cell culture and cell growth analysis

ESCC cells were cultured in Dulbecco's Modified Eagle Medium containing 10% fetal bovine serum. For colony formation experiments, the cells were treated with various concentrations of antitumor agent (Supplemental Table 2) for 48 h, then harvested and seeded in 6-well plates (2 × 10^3^ cells/well); the colony formation assay was performed as previously described 16, 17. Optical density at 546 nm (OD_546_) was measured and used to calculate colony formation rate according to the following formula: [(OD_546_ of the control group) - (OD_546_ of the treatment group)]/(OD_546_ of the control group)] × 100%. The results were used to determine half-maximal inhibitory concentration (IC_50_) values 18, 19.

### Western blotting

Total protein was extracted from ESCC cells according to a published protocol 20-22. Non-tumor Het-1A human esophageal epithelial cells or patient-derived ESCC cells expressing low levels of endogenous OCT1 were transfected with lentivirus particles harboring the full-length *OCT1* sequence or a siRNA targeting *OCT1*. OCT1 protein level was detected by western blotting (antibody from Abcam, Cambridge, UK; ab51363), with GAPDH serving as a loading control.

### Xenograft tumor model

All experiments with mice were approved by the Institutional Animal Care and Use Committee of Zhengzhou University (Henan, China) and were performed in accordance with the 1986 UK Animals (Scientific Procedures) Act and associated guidelines. To examine the growth of ESCC cells *in vivo*, we established a subcutaneous xenograft tumor model in nude mice. Cultured ESCC cells were subcutaneously injected into the mice 23, 24 and 3 or 4 days later, the mice were orally administered cisplatin (Supplemental Table 3) once every 2 days. After 3 weeks (10 treatments), tumors were harvested from the mice and tumor volume was calculated as (tumor length × tumor width × tumor width) / 2 25, 26. Tumor weight was measured using a precision balance. The rate of inhibition of tumor growth was calculated as [(tumor volume of control group) - (tumor volume of drug treatment group)] / (tumor volume of control group) × 100%; or as [(tumor weight of control group) - (tumor weight of drug treatment group)] / (tumor weight of control group) × 100%.

### Statistical analysis

Statistical analyses were performed with SPSS v9.0 software (SPSS Inc., Chicago, IL, USA). Differences between groups were evaluated for statistical significance by two-way analysis of variance with Bonferroni correction. *P* < 0.05 was considered significant. IC_50_ values of the tested agents against ESCC cells were calculated using Origin v6.0 software (OriginLab, Northampton, MA, USA).

## Results

### Expression of OCT transcription factors in ESCC

**Figure 1:**
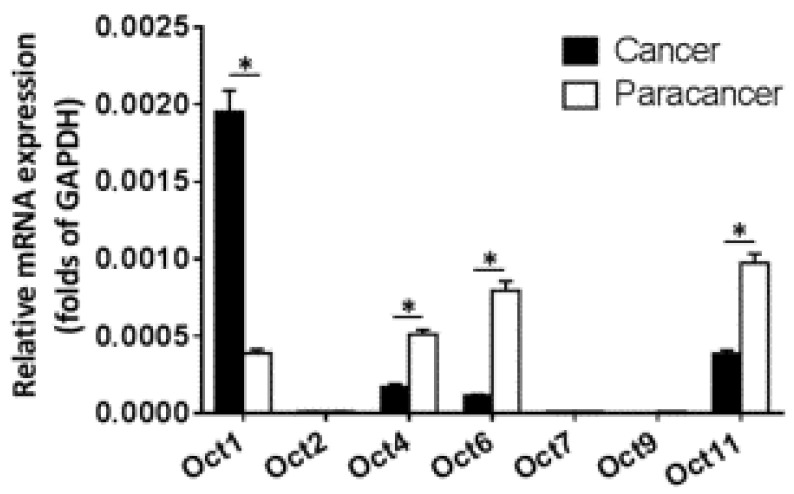
** Expression levels of OCT transcription factors in ESCC and paired non-tumor tissue**. The mRNA levels of OCT transcription factors was determined by qPCR. Results are shown as mean ± SD. *P < 0.05.

The expression of OCT transcription factors in ESCC and paired non-tumor tissue was examined by qPCR. *OCT2*, *OCT7*, and *OCT9* were not expressed in either type of sample (Figure 1). Meanwhile, *OCT1* was upregulated whereas *OCT4*, *OCT6*, and *OCT11* were downregulated in ESCC relative to non-tumor tissue, with a statistically significant difference observed for *OCT1* (*P* < 0.05).

### Methylation status of OCT gene promoters in ESCC

**Figure 2:**
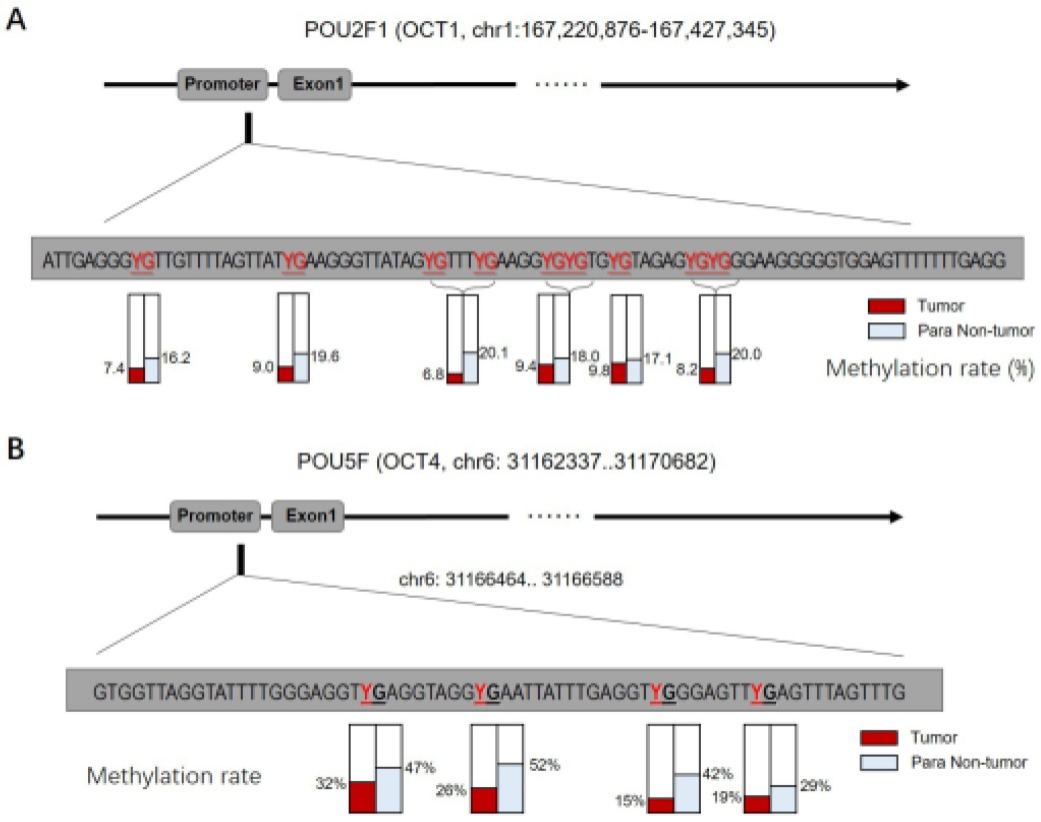
** The CpG sites or the methylation rates of OCTs transcription factor's promoter region in ESCC clinical specimens**. The CPG sites that could be methylated was shown as “YG” in Figure. There are four CpG sites in the selected region in the promoter of OCT1 (A) or OCT4 (B), and the methylation rates of these four sites in ESCC specimens or the non-tumor tissues was shown.

**Figure 3:**
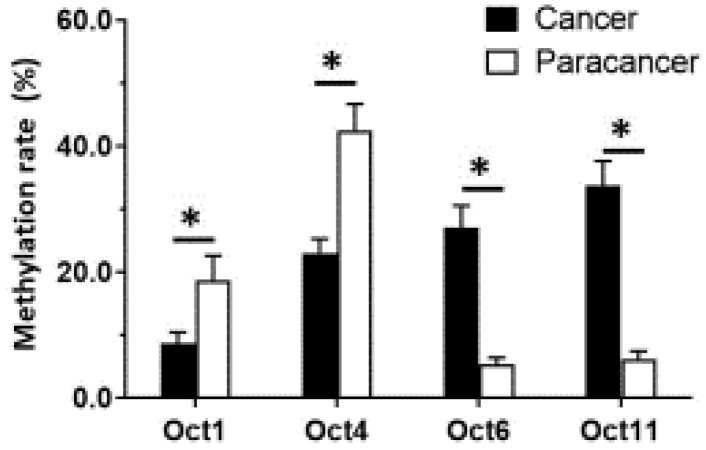
** The methylation rates of OCTs transcription factors**. The methylation rates of OCTs transcription factors OCT1, OCT4, OCT6 or OCT11 in ESCC specimens or the paired non-tumor specimens were shown as mean±SD. *P < 0.05 versus the methylation rates of OCTs transcription factors in ESCC clinical specimens or the paired non-tumor specimens.

Given that *OCT1*, *OCT4*, *OCT6*, and *OCT11* expression was dysregulated in ESCC specimens, we examined the methylation status of their promoters (Figure 2 and Table 1). Table 1 indicated the methylation rate data of each CpG site in the promoter regions of OCT1, OCT4, OCT6 and OCT11; whereas Figure 2 indicated the structure of the OCT1, OCT4 promoter region and the CpG sites in the selected region. The methylation rates at the *OCT1* and *OCT4* promoters were lower in ESCC (8.6% and 23.0%, respectively) compared to non-tumor tissue (18.6% and 42.5%, respectively) (Figure 3 and Table 1). However, the opposite results were observed for *OCT6* and *OCT11* (5.4% and 6.2% in the non-tumor tissues, respectively vs 27.1% and 33.7% in ESCC tissues).

**Figure 4:**
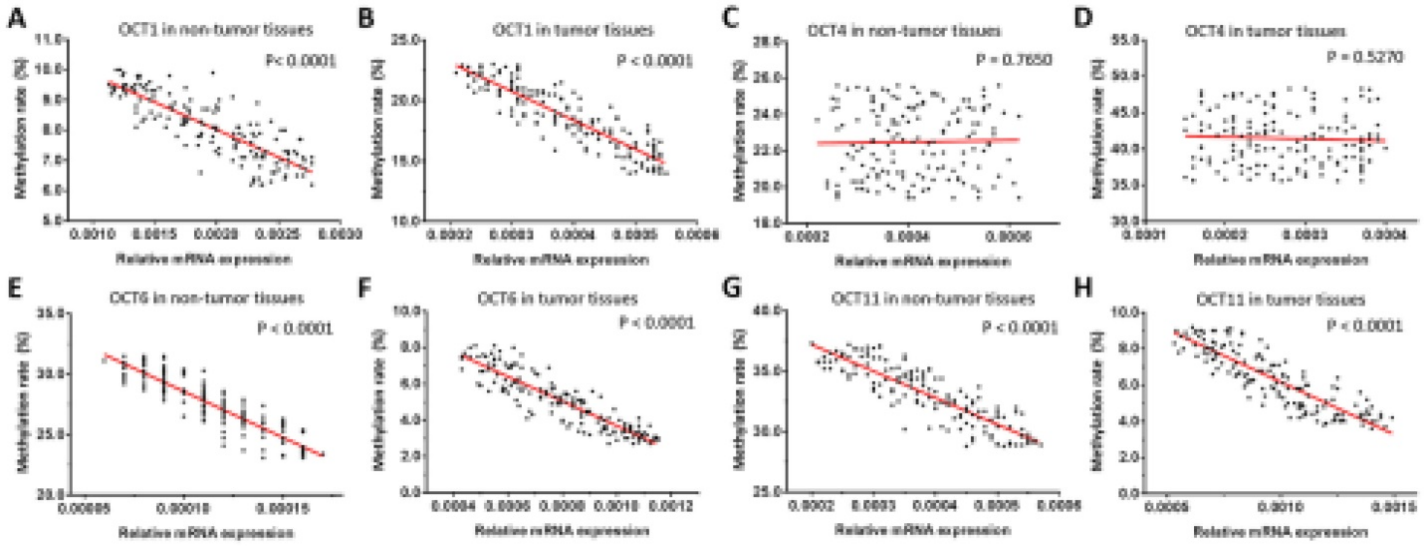
** Correlation between methylation rates of *OCT* promoter sequences and transcript levels in ESCC and non-tumor tissues**. (A-H) Correlations were determined by linear regression analysis and are shown as scatterplots for *OCT1* (A, B), *OCT4* (C, D), *OCT6* (E, F), and *OCT11* (G, H) in ESCC (B, D, F, H) and paired non-tumor (A, C, E, G) tissues. The Y-axis (ordinate) of the scatter-plot images is the methylation rates of OCTs transcription factor. The correlation between the methylation rates of OCTs transcription factors' promoter sequences and the expression level of OCTs in ESCC specimens or the paired non-tumor tissues was examined by linear regression and the P-values were shown. *P < 0.05.

We next examined the relationship between the expression levels (mRNA level) of OCT transcription factors and methylation of their gene promoters in ESCC and non-tumor tissues. We found that the methylation rates of *OCT1*, *OCT6*, and *OCT11* were negatively correlated with transcript levels in both types of sample (Figure 4). However, there was no association between promoter methylation status and expression level of *OCT4*. These results imply that the decreased methylation of *OCT1*, *OCT6*, and *OCT11* promoters, but not OCT4, is responsible for the observed dysregulation of these genes in ESCC tissue.

### Correlation between OCT1 and OCT4 expression and clinicopathologic characteristics of ESCC

**Figure 5:**
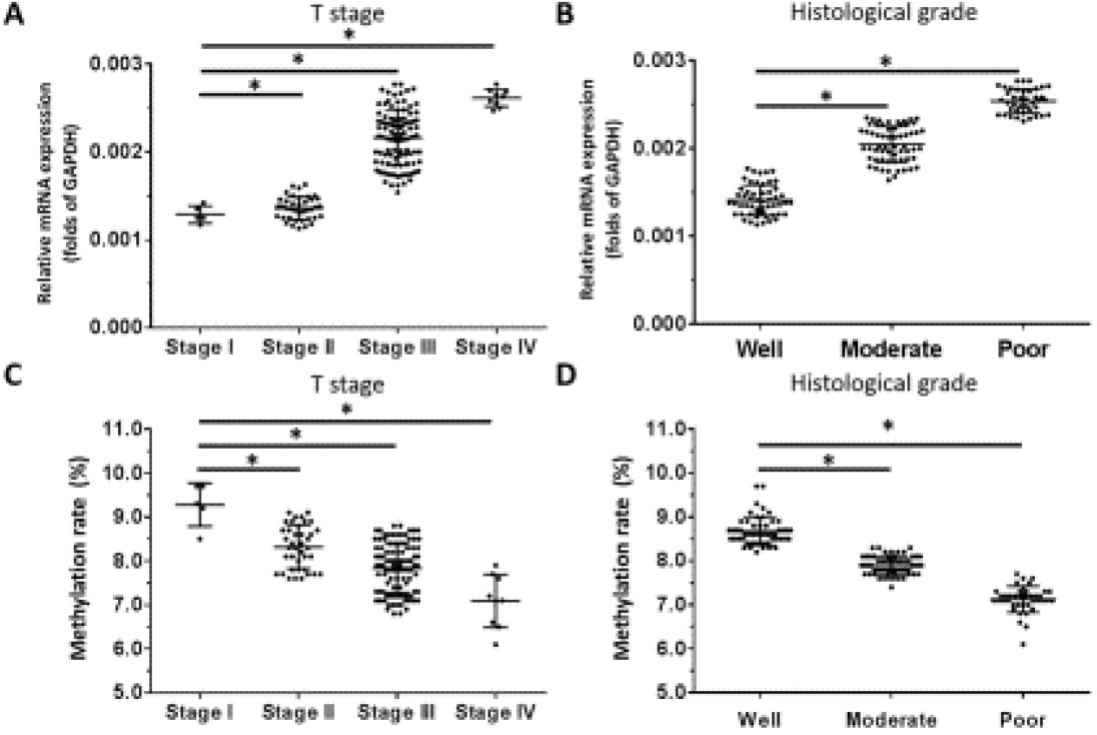
***OCT1* expression level and promoter methylation rate in ESCC**. *OCT1* level was detected by qPCR and *OCT1* promoter methylation rate was determined by BSP and NGS. (A-D) Transcript level and promoter methylation rate of *OCT1* in ESCC clinical specimens of different T stages (A, C) and histologic grades (B, D). *P < 0.05.

Based on our observation that *OCT1* was the most highly expressed OCT transcription factor in ESCC specimens and our previous finding that higher levels of *OCT4* were significantly associated with higher tumor grade in ESCC 10, we examined the correlation between *OCT1* and *OCT4* expression and clinicopathologic characteristics of the tumors specimens. We found that OCT1 levels were elevated for higher (more aggressive) T stages of ESCC (ie, Stage III or IV) compared to lower T stages (ie, Stage I or II) (Figure 5A). Moreover, high *OCT1* expression was significantly associated with higher histologic grade of ESCC (Figure 5B) and poor histologic differentiation (Figure 5B), a trend that was opposite to that of promoter methylation rate (Figure 5C, D).

**Figure 6:**
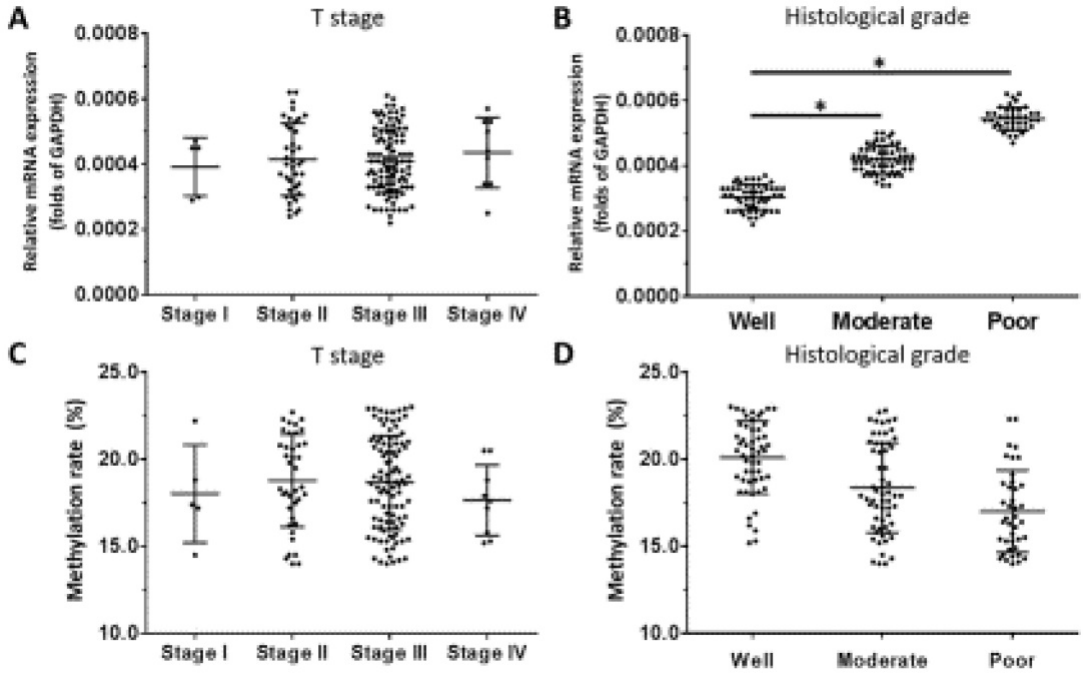
***OCT4* expression level and promoter methylation rate in ESCC**. *OCT4* level was detected by qPCR and *OCT1* promoter methylation rate was determined by BSP and NGS. (A-D) Transcript level and promoter methylation rate of *OCT4* in ESCC clinical specimens of different T stages (A, C) and histologic grades (B, D). *P < 0.05.

For *OCT4*, there was no relationship between mRNA level and T stage of ESCC (Figure 6A), although a positive association was observed with histologic grade (Figure 6B). *OCT4* expression was higher in poorly differentiated ESCC compared to well- or moderately differentiated samples (Figure 6B). There was no association between the methylation status of the *OCT4* gene promoter and T stage or histologic grade (Figure 6C, D).

### OCT1 regulates ESCC growth

We used ESCC cell lines to examine the function of OCT1 in ESCC. *OCT1* was highly expressed in KYSE70, KYSE140, and KYSE450 ESCC cell lines and three patient-derived cell lines (Nos. 1-3). In contrast, Het-1A, non-tumor human esophageal epithelial cells, and two other patient-derived ESCC cell lines (Nos. 4 and 5) expressed a low level of endogenous *OCT1*. We used KYSE70, KYSE140, and KYSE450 cells and patient-derived ESCC cell line Nos. 1-3 for *OCT1* knockdown and patient-derived ESCC cell line Nos. 4 and 5 for *OCT1* overexpression experiments (Supplemental Figure 1).

**Figure 7:**
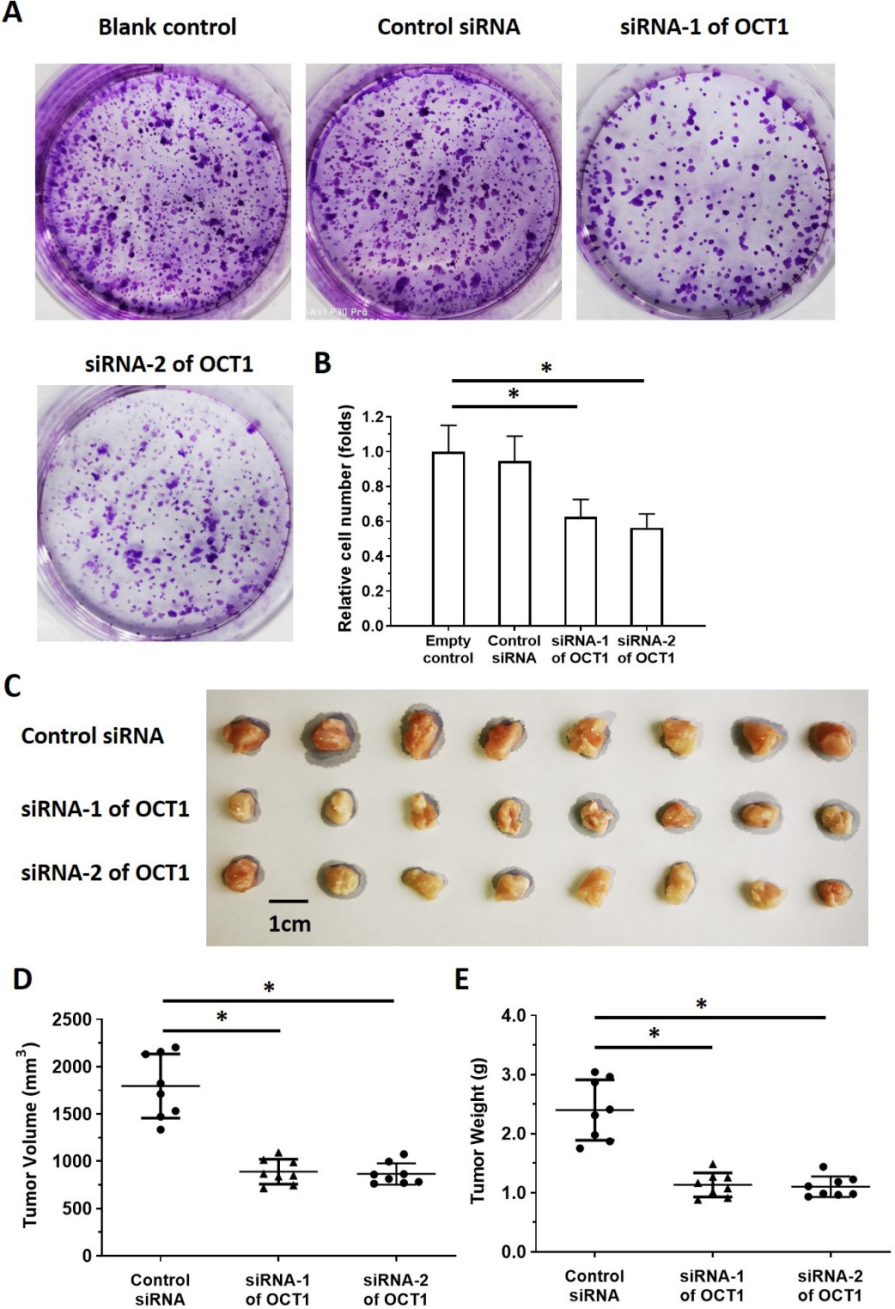
***OCT1* silencing inhibits proliferation in ESCC cells with high endogenous levels of OCT1.** (A, B) KYSE70 ESCC cells were transfected with *OCT1* siRNAs (siRNA-1 or siRNA-2) or a scrambled control siRNA, and colony formation was quantified. (C, D) Transfected cells were injected into nude mice and tumor volume (D) and weight (E) were recorded. Representative images of xenograft tumors are shown (C). *P < 0.05.

To clarify the role of OCT1 in ESCC cell survival, we knocked down *OCT1* expression and examined cell growth *in vitro* using ESCC cell lines and patient-derived cells expressing high endogenous levels of OCT1 (Supplemental Table 4). Additionally, we overexpressed *OCT1* in Het-1A cells and patient-derived cells (No. 4 and 5) with low endogenous OCT1 expression (Supplemental Figure 2). *OCT1* knockdown decreased proliferation in KYSE70, KYSE140, and KYSE450 cells and patient-derived ESCC cell line Nos. 1-3 (Figure 7, Supplemental Table 4), whereas *OCT1* overexpression in Het-1A cells and patient-derived ESCC cell line Nos. 4 and 5 had the opposite effect (Supplemental Figure 2).

### OCT1 silencing enhances the sensitivity of ESCC cells to antitumor agents

It was previously reported that OCT1 modulates drug resistance in prostate cancer cells. To test whether this extends to ESCC, we used a mouse xenograft model treated with the antitumor agents cisplatin, 5-FU, and paclitaxel. Drug treatment reduced ESCC cell colony formation in a dose-dependent manner, whereas *OCT1* knockdown via siRNA-1 or siRNA-2 enhanced the antitumor effect of cisplatin (Supplemental Figure 3A, B and Table 2). The IC_50_ values for KYSE70, KYSE140, and KYSE450 cells and three patient-derived ESCC cell lines with high endogenous OCT1 expression are shown in Table 2.

We next examined the *in vivo* activity of antitumor agents using a mouse xenograft tumor model. Treatment with cisplatin inhibited the growth of subcutaneous ESCC-cell derived tumors (Supplemental Figure 3C, D). On the other hand, tumors derived from *OCT1*-depleted cells showed enhanced the sensitivity to antitumor agents (Supplemental Figure 3C-E), as evidenced by the decreased IC_50_ values (Table 3).

To further examine the role of OCT1 in drug resistance in ESCC, patient-derived ESCC cell line Nos. 4 and 5 with low endogenous levels of OCT1 were transfected with an *OCT1* overexpression construct and treated with cisplatin, 5-FU, and paclitaxel. OCT1 overexpression enhanced the resistance of ESCC cells to antitumor agents, as evidenced by increased IC_50_ values (Supplemental Table 5). Thus, the sensitivity of ESCC cells to antitumor agents is enhanced by *OCT1* silencing whereas *OCT1* overexpression has the opposite effect.

## Discussion

ESCC is one of the most aggressive neoplasms and has poor clinical outcome 27, 28 that is partly attributable to the inherent resistance of ESCC cells to chemotherapy 29, 30. It is important from a clinical standpoint to clarify the mechanisms underlying this resistance so that more effective treatments can be developed. In the present work, we determined that *OCT1* was more highly expressed in ESCC relative to non-tumor tissue, whereas the opposite trend was observed for *OCT4*, *OCT6*, and *OCT11*. The results of the BSP and NGS experiments revealed that *OCT1*, *OCT6*, and *OCT11* levels were negatively correlated with the methylation rate of their promoters, with a much lower rate observed for *OCT1* than for *OCT6* and *OCT11*. These results indicate that the altered methylation status may potentially participate in the dysregulated expression of these OCTs (*OCT1*, *OCT6* or *OCT11*) in ESCC and the mechanism of *OCT4*'s expression in ESCC needed for the further research. OCT1 may be a useful therapeutic target for ESCC treatment. However, functional redundancy among OCT proteins could contribute to the failure of single-target inhibitors; it is therefore worthwhile to determine the expression levels of all OCT family members.

There have been few studies to date investigating the role of OCT1 in ESCC 31, 32. OCT1 was shown to act as a positive regulator of ESCC cells in conjunction with signal transducer and activator of transcription 31. It was also suggested that high OCT1 expression enhances drug resistance in prostate cancer cells 32. Therefore, OCT1 could function as an important regulator for cancer cells and the drug-resistance, however, the detailed of OCT1 function is still not very clearly. In prostate carcinoma cells, OCT1 could function by interacting with GATA binding protein 2 (GATA2), forkhead box A1 (FOXA1), or androgen receptor 32. Moreover, OCTs transcription factors have been considered to participate in mediating the self-renew or the stemness features of cancerous cells 10. In the present study, *OCT1* overexpression increased proliferation in Het-1A cells whereas *OCT1* knockdown in ESCC cell lines or patient-derived ESCC cells with high endogenous *OCT1* expression had the opposite effect. Thus, modulating the expression of OCT1 either directly or indirectly by targeting its epigenetic modifiers is a potential strategy for overcoming the drug resistance of ESCC cells.

We previously demonstrated that OCT4 was expressed in ESCC clinical specimens and that OCT4 level was unrelated to T stage but was positively associated with histological grade of ESCC 10. The results presented here are consistent with our earlier findings. Moreover, we found that *OCT1* expression or promoter methylation was related to both T stage and histological grade. OCT transcription factors are key regulators of the proliferation, metastasis, and drug resistance of human cancer cells and are therefore promising targets of antitumor drugs.

Methylation of gene promoters is a regulatory mechanism that is typically associated with transcriptional repression 33-36. For instance, hypermethylation of tumor suppressor gene promoters in tumor tissues results in the loss of gene expression, whereas hypomethylation of oncogene promoters leads to their aberrant activation 37-40. Thus, the methylation status of genes promoters has clinical relevance in the context of cancer. In this study, we evaluated the methylation rate of *OCT* gene promoters directly by BSP and NGS. In contrast, traditional methylation-specific PCR can only detect the presence or absence of methyl marks at CpG sites in single clones 41-43. Our method had greater efficiency as it allowed high-throughput screening of promoter regions. We also found that the rate of methylation of the *OCT4* promoter was lower in ESCC compared to non-tumor tissue but that this was unrelated to transcript expression. Additional studies are needed to clarify the clinical significance of this observation.

Moreover, the previous studies mainly used existing current cell lines, but recent studies have shown that tumor cells are affected by tumor microenvironment and other factors in tumor tissues, which are different from current cell lines cultured *in vitro* for a long time 44. The cell lines may not reflect the actual condition of the cells in the patient's tumor tissue 44. A feasible strategy to solve this problem is to obtain and use PDCs, and there have been many reports on related studies 45-48. Our research not only used three ESCC cell lines, but also established patient-derived cell lines as a supplement. This not only makes the results more credible, but also has guiding significance for related research.

## Conclusion

Changes in promoter methylation status are responsible for the altered expression of *OCT1*, *OCT6*, and *OCT11* in ESCC tissue. These results indicate that targeting these proteins or the factors regulating their methylation/demethylation - especially that of OCT1 - may be an effective strategy for the treatment of ESCC.

## Supplementary Material

Supplementary figures and tables.

## Figures and Tables

**Table 1:** The CpG sites or the methylation rates of OCTs transcription factor's promoter region in ESCC clinical specimens

Targets	Sequence (5'-3')	Methylation rate (%)	
		Tumor	Non-tumor
OCT1	ATTGAGGG**YG**TTGTTTTAGTTAT**YG**AAGGGTTATAG**YG**TTT**YG**AAGG**YGYG**TG**YG**TAGAG**YGYG**GGAAGGGGGTGGAGTTTTTTTGAGG	8.60%	18.60%
OCT4	GTGGTTAGGTATTTTGGGAGGT**YG**AGGTAGG**YG**AATTATTTGAGGT**YG**GGAGTT**YG**AGTTTAGTTTG	23.00%	42.50%
OCT6	T**YG**AGATTTTTTTTTTTTGGAATTT**YG**GAG**YGYG**GT**YG**GGTATTAGGGGTT**YGYG**TAGGATTGTAGAAT**YG**TT	27.10%	5.40%
OCT11	TTGTAATTTTAGGGAAGTTTAATTGAAGTTTGATT**YG**T**YG**TTTTAGTATTTAATTAGGGATAAGAGAATTTGAG	33.70%	6.20%

YP: the methylation sites (CpG sites) of the OCTs transcription factor's promoter region.

**Table 2:** The *IC_50_* values of agents on ESCC cells' colony formation

Cell lines	Agents	control siRNA	siRNA of OCT1	siRNA-2 of OCT1
		*IC_50_* values of agents (nmol/L)		
KYSE70	Cisplatin	186.33±12.75	54.35±7.70	60.30±11.25
	5-Fu	490.90±17.83	99.80±10.45	122.15±18.31
	Paclitaxel	31.92±2.74	2.90±0.88	13.92±7.50
KYSE140	Cisplatin	242.42±22.15	105.76±6.79	83.26±3.33
	5-Fu	1.17±0.48 (μmol/L)	230.61±12.57	333.67±28.93
	Paclitaxel	120.07±14.49	20.58±5.44	35.42±6.03
KYSE450	Cisplatin	175.39±27.53	47.22±9.66	44.26±7.88
	5-Fu	412.99±26.75	12.36±3.50	75.62±10.36
	Paclitaxel	86.24±9.88	12.40±7.21	20.95±6.67
PDCs No. 1	Cisplatin	104.67±8.52	21.43±4.69	28.45±5.60
	5-Fu	226.99±45.21	76.19±6.54	65.47±5.14
	Paclitaxel	135.01±35.85	51.25±6.11	79.34±8.69
PDCs No. 2	Cisplatin	129.35±4.30	86.60±7.81	48.11±15.73
	5-Fu	728.95±38.21	320.88±46.67	239.85±71.70
	Paclitaxel	285.85±84.49	120.00±37.28	98.67±9.75
PDCs No. 3	Cisplatin	122.65±10.33	20.88±4.10	40.88±14.59
	5-Fu	374.48±72.76	59.33±33.48	92.68±20.72
	Paclitaxel	119.13±20.94	27.47±3.42	35.01±2.49

PDCs: Patients-derived cells.

**Table 3:** The *IC_50_* values of Cisplatin on ESCC cells' subcutaneous growth

Cell lines		control siRNA	siRNA of OCT1
		*IC_50_* values of Cisplatin (mg/kg)	
KYSE70	Tumor volume	0.43±0.03	0.16±0.06
	Tumor weights	0.37±0.10	0.11±0.02
KYSE140	Tumor volume	0.38±0.05	0.10±0.01
	Tumor weights	0.53±0.12	0.20±0.03
KYSE450	Tumor volume	0.70±0.20	0.24±0.04
	Tumor weights	0.59±0.24	0.16±0.04
PDCs No. 1	Tumor volume	0.33±0.05	0.24±0.08
	Tumor weights	0.30±0.01	0.18±0.02
PDCs No. 2	Tumor volume	0.60±0.12	0.20±0.06
	Tumor weights	0.75±0.08	0.25±0.10
PDCs No. 3	Tumor volume	0.39±0.22	0.11±0.03
	Tumor weights	0.52±0.101	0.28±0.14

PDCs: Patients-derived cells.

## Abbreviations

OCTs: The Pit-Oct-Unt transcription factors/Octamer transcription factors
ESCC: esophageal squamous cell carcinoma
BSP: bisulfite genomic sequence
NGS: next generation sequencing
MSP: methylmion specific PCR
