# Supplementary material for: DNA methylation integratedly modulates the expression of Pit-Oct-Unt transcription factors in esophageal squamous cell carcinoma
Source: J Cancer. 2021 Jan 15;12(6):1634–43. doi: 10.7150/jca.49231 (PMC7890322; doi:10.7150/jca.49231)
Supplement: Supplementary file 1 — Supplementary figures and tables. [file jcav12p1634s1.pdf]

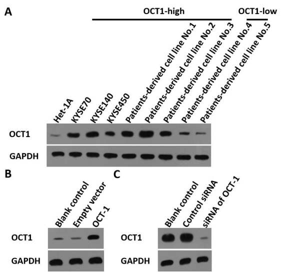


**Supplemental Figure 1. Endogenous *OCT1* levels in ESCC cell lines.** (A–C) Het-1A, a non-tumor cell line, along with the ESCC cell lines KYSE70, KYSE140, and KYSE450 and five patient-derived ESCC cell lines (Nos. 1–5) were cultured and analyzed by western blotting for OCT1 expression (A). Cell lines with low endogenous levels of OCT1 (B) were used for *OCT1* overexpression experiments whereas those with high endogenous levels were used for siRNA-mediated *OCT1* gene silencing (C).

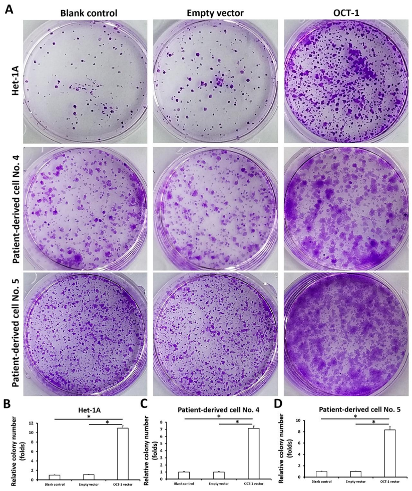


**Supplemental Figure 2. *OCT1* overexpression enhances proliferation in ESCC cells with low endogenous levels of OCT1**. (A–D) Het-1A cells and patient-derived ESCC cell line Nos. 4 and 5 were transfected with an *OCT1* overexpression construct or empty vector. Representative images of cells (A) and quantitative analysis of colony formation (B–D) are shown. Results are expressed as mean ± SD. $^{*}P < 0.05$.

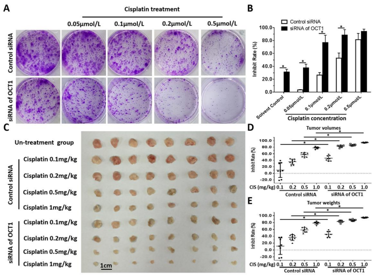


**Supplemental Figure 3 *OCT1* silencing enhances the sensitivity of ESCC cells to antitumor agents**. (A–D) KYSE70 ESCC cells were transfected with *OCT1* siRNA or scrambled control siRNA and cultured for assessment of in vitro colony formation (A, B) or injected into nude mice to evaluate subcutaneous tumor growth (C, D). For in vitro experiments, cells were treated with the indicated concentrations of cisplatin. Representative images of colonies are shown (A) and growth inhibition was quantified with the colony formation assay (B). Results are shown as mean ± SD. For in vivo experiments, mice were orally administered the indicated doses of cisplatin. Representative images of subcutaneous tumors are shown (C) along with rates of ESCC cell-derived tumor growth inhibition by antitumor agents (D, E). *$P < 0.05$ (n = 10).

**Supplemental Table 1. The baseline information of patients in the presence work**

| Parameters | Cases |
|---|---|
| Gender | |
| Male | 98 |
| Female | 52 |
| Location | |
| Middle | 109 |
| Lower | 41 |
| Lymph node metastasis | |
| - | 54 |
| + | 96 |
| UICC stage | |
| I | 5 |
| II | 39 |
| III | 97 |
| IV | 9 |
| Histological grade | |
| Well | 52 |
| Moderate | 58 |
| Poor | 40 |

**Supplemental Table 2. The concentration of antitumor agents, Cisplatin, 5-Fu or Paclitaxel, used in cell-based experiments**

| Agents | Concentration (μmol/L) | | | |
|---|---|---|---|---|
| Cisplatin | 0.5 | 0.2 | 0.1 | 0.05 |
| 5-Fu | 2.0 | 1.0 | 0.5 | 0.2 |
| Paclitaxel | 0.2 | 0.1 | 0.05 | 0.02 |

**Supplemental Table 3. The concentration of Cisplatin used in animal experiments**

| Agent | Concentration (mg/kg) | | | |
|---|---|---|---|---|
| Cisplatin | 1 | 0.5 | 0.2 | 0.1 |

**Supplemental Table 4 Inhibition rates of OCT1 siRNA on cells' colony formation.**

| Cell lines | Blank control | control siRNA | siRNA of OCT1 |
|---|---|---|---|
| | Inhibition rates (%) | | |
| KYSE70 | - | - | 41.00±4.10 |
| KYSE140 | - | - | 37.08±7.65 |
| KYSE450 | - | - | 27.72±5.15 |
| PDCs No. 1 | - | - | 35.43±3.17 |
| PDCs No. 2 | - | - | 57.92±5.45 |
| PDCs No. 3 | - | - | 43.08±4.80 |

PDCs: Patients-derived cells

**Supplemental Table 5 The effect of OCT1 overexpression on agents' *IC₅₀* values**

| Cell lines | Agents | control siRNA | siRNA of OCT1 |
|---|---|---|---|
| | | $IC_{50}$ values of agents (nmol/L) | |
| PDCs No. 4 | Cisplatin | 88.90±9.59 | 369.72±38.97 |
| | 5-Fu | 153.71±49.94 | 687.35±65.40 |
| | Paclitaxel | 58.60±10.77 | 390.83±25.65 |
| PDCs No. 5 | Cisplatin | 96.67±40.18 | 459.51±54.27 |
| | 5-Fu | 260.59±16.67 | 1.56±2.34 (μmol/L) |
| | Paclitaxel | 45.60±4.34 | 160.00±17.53 |

PDCs: Patients-derived cells
